# Enhancing Histological Visualization of Tooth and Bone Ground Sections Using *Terminalia chebula* Stain: A Comparative Analysis

**DOI:** 10.1155/tswj/5896675

**Published:** 2026-06-18

**Authors:** Sangeetha Ramu, Yashika S. Shinde, Vidyadevi Chandavarkar

**Affiliations:** ^1^ Department of Oral and Maxillofacial Pathology, Dayananda Sagar College of Dental Sciences, Rajiv Gandhi University of Health Sciences, Bengaluru, India, rguhs.ac.in; ^2^ Department of Oral and Maxillofacial Pathology and Oral Microbiology, School of Dental Sciences, Sharda University, Greater Noida, Uttar Pradesh, India, sharda.ac.in

**Keywords:** bone histology, ground section, natural stain, *Terminalia chebula*, tooth histology

## Abstract

**Background:**

Visualization of mineralized tissues such as tooth and bone in ground sections is widely used for histological studies. However, unstained ground sections often provide limited contrast and obscure microscopic details. Natural plant‐based stains have been explored as cost‐effective and safer alternatives to synthetic dyes. *Terminalia chebula*, rich in tannins and polyphenolic compounds, has shown potential staining affinity for hard tissues.

**Objectives:**

The aim of this study is to evaluate the staining efficacy of *T. chebula* on ground sections of tooth and bone by comparing stained and unstained sections, and to assess contrast, structural detail, and observer acceptance under stereomicroscope and compound microscope.

**Methods:**

Forty extracted normal teeth and 40 bone samples from human skulls were prepared into thin ground sections (~50 *μ*m). Each section was initially observed unstained (Group 2) and then stained with *T. chebula* extract for 4 h (Group 1). Photomicrographs were taken under both microscopes and evaluated by blinded oral pathologists and students for contrast, structural detail, and acceptance using standardized grading and scoring systems.

**Results:**

*T. chebula* staining significantly improved contrast in tooth sections under both stereomicroscope and compound microscope (*p* < 0.001). Enhanced visualization of enamel rods, dentinal tubules, and cementum was observed. Bone sections demonstrated improvement in contrast under stereomicroscopy; however, under compound microscopy, unstained bone sections showed relatively higher Grade 1 contrast. Improvements in structural detail were more pronounced in tooth sections than bone sections. Observer acceptance was significantly higher for stained tooth sections (*p* < 0.001), whereas moderate but significant improvement was noted for bone sections.

**Conclusion:**

*T. chebula* stain enhances contrast and improves visualization of microstructural features in tooth ground sections, supporting its potential as an effective and economical adjunct for teaching and research in dental histology. Further studies are required to optimize staining protocols and to evaluate its broader applicability, particularly in bone specimens.

## 1. Introduction

Ground section of hard tissue is a widely used method to study the histology of mineralized structures under a microscope. The hard tissue components typically examined in ground sections include enamel, dentin, cementum, and bone. Visualization of these structures depends on the degree of light passing through their crystalline structure [1]. However, unstained ground sections often produce obscure images, limiting the clarity of histological details [2].

Histological staining enhances contrast and depth perception, facilitating better interpretation of tissue architecture [1]. The effectiveness of a stain depends on its chemical composition, affinity for tissue components, and interaction with mineralized matrices [3, 4] Conventionally, decalcified sections stained with hematoxylin and eosin or other special stains are used for detailed histological evaluation [5]. Although these methods provide excellent cellular detail, they require decalcification, which may alter or remove the natural mineral architecture of hard tissues. In contrast, ground sections preserve the mineralized structure but lack adequate contrast, highlighting the need for effective staining methods specifically for ground sections.

*Terminalia chebula*, a plant rich in tannins and polyphenolic compounds, exhibits a strong affinity for proteinaceous and mineralized tissue components, supporting its potential as a natural histological stain. Previous studies have reported its ability to enhance contrast in dental ground sections, indicating its possible application in hard tissue histology [6]. However, existing literature is limited, and there is a lack of systematic evaluation of its efficacy across different mineralized tissues and imaging modalities.

Compared with conventional synthetic stains, plant‐based stains such as *T. chebula* offer potential advantages including low cost, ease of preparation, and reduced toxicity, making them attractive alternatives, especially in educational and resource‐limited settings. Nevertheless, their staining characteristics, consistency, and applicability require further validation. Unlike previous studies that have primarily focused on dental tissues, the present study provides a comparative evaluation of *T. chebula* staining in both tooth and bone ground sections, along with assessment under different microscopic modalities and inclusion of observer‐based parameters such as contrast, structural detail, and acceptance.

Traditionally, undergraduate, postgraduate, and research scholars have studied the microscopic structural details of mineralized teeth and bone using unstained ground sections. To the best of our knowledge, few studies have investigated staining ground sections, and stained ground sections are not routinely used in teaching and training of hard tissue pathology.

Therefore, the present study is aimed at evaluating the staining efficacy of *T. chebula* on ground sections of tooth and bone and to compare stained and unstained sections in terms of contrast, structural detail, and observer acceptance under compound and stereomicroscopes. By doing so, this study seeks to address the existing gap in the application of natural stains for ground sections and to assess their potential utility in dental histology.

## 2. Materials and Methodology

### 2.1. Source of Data

The study samples were collected from the Department of Oral and Maxillofacial Surgery and the Department of General Anatomy, DSCDS, Bengaluru.

The study was approved by the Institutional Ethics Committee (IEC No: 187‐IRB‐2023).

### 2.2. Criteria for Selection

#### 2.2.1. Inclusion Criteria

The inclusion criteria are listed as follows:

• Extracted premolar obtained following routine orthodontic procedures.
• Bone specimens were obtained from the cut pieces of the study skull model.

#### 2.2.2. Exclusion Criteria

The exclusion criteria are listed as follows:

• Extracted teeth exhibiting caries, restorations, fractures, developmental anomalies, or any pathological changes.
• Skull bone specimens exhibiting pathological changes, defects, or structural damage that could affect the study outcomes.

Variations in tooth type and age may influence structural visibility; however, in this study, each specimen was evaluated in both unstained and stained conditions, thereby serving as its own control. This within‐sample comparison minimized the influence of such variations on the overall findings.

### 2.3. Infection Control Protocols

The collection, storage, sterilization, and handling of extracted teeth used in this study adhered to the guidelines and recommendations of the Occupational Safety and Health Administration (OSHA) and the Centers for Disease Control and Prevention (CDC) [7].

Bone pieces we have taken from the study skull model and were handled in accordance with institutional anatomical and ethical guidelines.

#### 2.3.1. Study Setting

All procedures were conducted in a controlled laboratory environment.

#### 2.3.2. Materials and Armamentarium

The materials and armamentarium are listed as follows:

• Dried *T. chebula* seeds
• Amber‐colored storage bottles
• Mortar and pestle
• Distilled water
• Filter paper
• Microscope slides and cover slips
• Compound microscope
• Stereomicroscope
• DSLR camera

#### 2.3.3. Sample Size Calculation

Sample size estimation was performed using G∗Power 3.0.10 software with the following parameters:

• Statistical test: Chi‐square (*χ* ^2^) goodness‐of‐fit test. Chi‐square goodness‐of‐fit test was selected as the study involved categorical grading (contrast, structural detail, acceptance).
• Effect size: 0.5.
• Power (1‐*β*): 0.80.
• Total sample size: 39 (rounded to 40).

The effect size of 0.5 was considered a moderate effect based on standard statistical conventions in the absence of prior comparable studies. A total of 40 teeth and 40 bone specimens were included and evaluated individually. Each specimen served as its own control (before and after staining), thereby reducing intersample variability.

### 2.4. Study Method

The study comprised 40 tooth specimens and 40 bone pieces. Ground sections of teeth were prepared, and photomicrographs were captured under both compound and stereomicroscopes. The same specimens were then stained using *T. chebula* stain, mounted, and photomicrographs of the identical areas were taken again under both microscopes. This procedure was similarly applied to bone ground sections. Both the stereomicroscope and compound microscope were used to evaluate staining efficacy at different magnification levels, allowing assessment of both overall architecture and finer structural details.

Photomicrographs were captured at standardized magnifications for all specimens to ensure consistency in evaluation. Blinded oral pathologists and students evaluated the photomicrographs to assess staining efficacy, structural detail, and acceptance level. A total of two oral pathologists and two students participated in the evaluation. Prior to assessment, evaluators were oriented and calibrated using standardized criteria to ensure consistency in scoring:

• Group 1: Ground sections stained with *T. chebula.*
• Group 2: Unstained ground sections (control).

### 2.5. Preparation of *T. chebula* Stain

**Figure 1:**
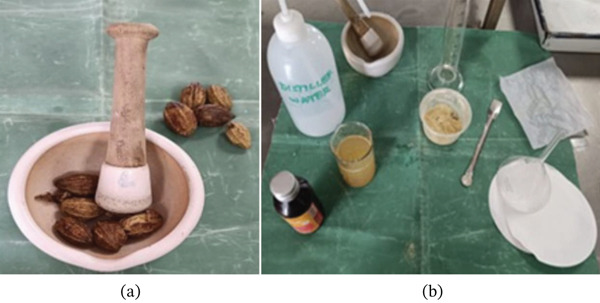
(a and b) Collection and preparation of *Terminalia chebula* stain.

*T. chebula* seeds were procured from the local market and crushed into powder using a mortar and pestle. Approximately 4 g of powder was dissolved in 100 mL distilled water and filtered through filter paper. The prepared stain was stored in amber‐colored bottles at room temperature, with a shelf life of 24 months [6] (Figure 1a,b). Seeds were selected based on their higher tannin and polyphenolic content, which are known to enhance staining affinity for mineralized tissues. The pH of the prepared stain was not specifically standardized; however, preparation was performed using a consistent protocol to maintain uniformity across samples.

### 2.6. Preparation of Ground Sections and Staining Procedure

#### 2.6.1. Tooth Sections

Ground sections of teeth were prepared following a standard protocol. Teeth were immersed in hydrogen peroxide solution for 24 h, rinsed with sterile water, and ground using a carborundum stone.

Ground sections were prepared to an approximate thickness of 50 *μ*m using a standardized manual grinding technique. Uniformity of thickness was ensured by a consistent grinding protocol and periodic microscopic verification of translucency.

Sections were cleared in xylene for 1 min, hydrated through graded alcohol concentrations for 3–4 min, and rinsed in distilled water for 2–3 min.

• Step 1 (control group): Unstained sections were cleaned, dried, mounted on slides, and observed under compound and stereomicroscopes. Photomicrographs were taken and stored. Sections were then kept in distilled water.
• Step 2 (study group): The same sections were stained with *T. chebula* for 4 h. The staining duration of 4 h was selected based on preliminary standardization and available literature to ensure adequate stain penetration. Differentiation with 95% alcohol for approximately 15 sec was performed to remove excess stain and enhance contrast. Sections were dried, cleared with xylene, mounted with DPX, and observed under microscopes. Photomicrographs of the same areas were captured and stored.

### 2.7. Bone Sections

Cut pieces of bone from the skull model were prepared into ground sections following the same protocol as for teeth.

### 2.8. Evaluation

Blinded oral pathologists and students evaluated the photomicrographs to assess staining efficacy, structural detail, and acceptance level. The photomicrographs were coded and presented in a randomized manner, ensuring that assessors were unaware of whether the images belonged to stained or unstained groups. This minimized observer bias. Scores from both groups were combined for analysis, and interobserver agreement was not calculated.

The stained sections of teeth and bone specimens were evaluated for the quality of staining in terms of contrast and structural details and acceptance level among the oral pathologists and students; the following are the structural details of teeth and bone to be appreciated in ground section.

• Enamel: Enamel rods, tufts, lamellae, spindles, and incremental lines, gnarled enamel.
• Dentin: Dentinal tubules, dentin–enamel junction, primary dentin, secondary dentin, interlobular dentin, and Tome′s granular layer.
• Cementum: CEJ, cemento–dentinal junction, cellular and acellular cementum, incremental lines.
• Bone: Haversian canal, lamellae, osseous lacunae, Volkmann′s canal, canaliculi.

### 2.9. To Assess the Staining Efficacy

The grading of the unstained and stained ground section was done from Grade 1 to 3 according to the differentiation and contrast of tooth and bone.

a. Grade 1: Tissue brightly contrasted.
b. Grade 2: Tissues moderately distinguishable.
c. Grade 3: Tissues distinguishable with difficulty.

### 2.10. To Compare the Structural Details

The structural details of unstained and stained tooth and bone ground sections were assessed as follows:

a. Score 1: Structural details are well appreciated.
b. Score 2: Structural details are moderately appreciated.
c. Score 3: Structural details are poorly appreciated.

### 2.11. To Assess the Acceptance Level

Acceptance level among the participants in the study for using *T. chebula* as a staining agent for the ground section of tooth and bone was assessed using the following scale:

Henceforth, do you accept *T. chebula* staining agent to use for staining the ground section before observing under a microscope were compared with no staining?

a. Highly acceptable
b. Moderately acceptable
c. Slightly acceptable
d. Not acceptable

## 3. Results

All specimens (*n* = 40 tooth and *n* = 40 bone) were evaluated in *T. chebula*‐stained sections (Group 1) and unstained sections (Group 2). As each specimen was assessed before and after staining, comparisons represent paired observations. Statistical analysis was performed using the Chi‐square test for categorical data, and *p* < 0.05 was considered statistically significant.

### 3.1. Tooth Sections Under Stereomicroscope

**Figure 2:**
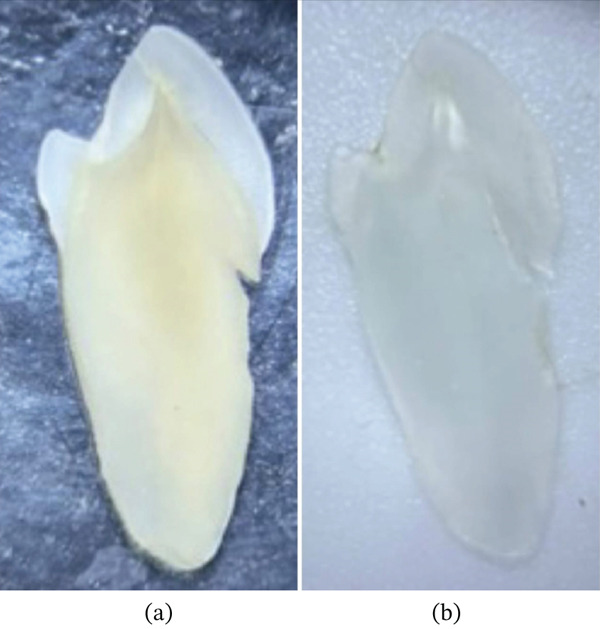
(a) Photomicrograph of *Terminalia chebula* stained tooth specimen under stereomicroscope. (b) Photomicrograph unstained tooth specimen under stereomicroscope.

A total of 40 tooth sections were evaluated in each group. In Group 1 (*T. chebula* stained) (Figure 2a), 29 samples (72.5%) demonstrated high contrast (Grade 1), compared with 13 samples (32.5%) in Group 2 (unstained) (Figure 2b). The difference in contrast between the groups was statistically highly significant (*p* < 0.001) (Table 1).

**Table 1:** Comparison of contrast, structural details, and acceptance levels in tooth sections under stereomicroscope.

Parameter	Category	Stained *n* (%)	Unstained *n* (%)	*p* value
Contrast	Grade 1	29 (72.5)	13 (32.5)	< 0.001
	Grade 2	11 (27.5)	19 (47.5)	
	Grade 3	0 (0)	8 (20)	
Structural details	Score 1	20 (50)	9 (22.5)	0.005
	Score 2	16 (40)	16 (40)	
	Score 3	4 (10)	15 (37.5)	
Acceptance level	Highly acceptable	18 (45)	10 (25)	<0.001
	Moderately acceptable	13 (32.5)	2 (5)	
	Slightly acceptable	9 (22.5)	22 (55)	
	Not acceptable	0 (0)	6 (15)	

Structural detail scores revealed that 20 samples (50%) in the stained group showed Score 1, compared with nine samples (22.5%) in the unstained group. A higher proportion of unstained samples (37.5%) exhibited Score 3 compared with the stained group (10%). This difference was statistically significant (*p* = 0.005) (Table 1).

Acceptance levels showed that 18 samples (45%) in the stained group were rated as highly acceptable, compared with 10 samples (25%) in the unstained group. A greater proportion of unstained samples were categorized as slightly acceptable (55%) and not acceptable (15%). The difference was statistically highly significant (*p* < 0.001) (Table 1).

### 3.2. Bone Sections Under Stereomicroscope

**Figure 3:**
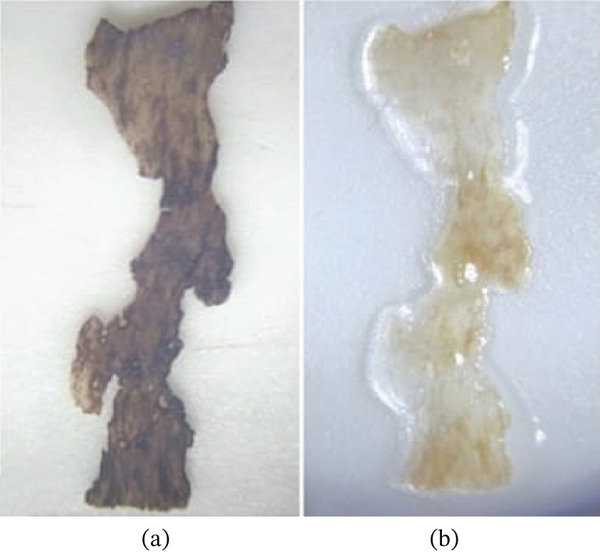
(a) Photomicrograph of *Terminalia chebula* stained bone specimen under stereomicroscope. (b) Photomicrograph unstained bone specimen under stereomicroscope.

In bone sections, seven samples (17.5%) in the stained group showed Grade 1 contrast, whereas none of the unstained samples demonstrated Grade 1 contrast (Figure 3a,b). A higher proportion of unstained samples (60%) showed Grade 3 contrast compared with the stained group (22.5%). The difference was statistically highly significant (*p* < 0.001) (Table 2).

**Table 2:** Comparison of contrast, structural details, and acceptance levels in bone sections under stereomicroscope.

Parameter	Category	Stained *n* (%)	Unstained *n* (%)	*p* value
Contrast	Grade 1	7 (17.5)	0 (0)	< 0.001
	Grade 2	24 (60)	16 (40)	
	Grade 3	9 (22.5)	24 (60)	
Structural details	Score 1	0 (0)	0 (0)	0.07
	Score 2	4 (10)	10 (25)	
	Score 3	36 (90)	30 (75)	
Acceptance level	Highly acceptable	0 (0)	0 (0)	< 0.003
	Moderately acceptable	7 (17.5)	7 (17.5)	
	Slightly acceptable	24 (60)	10 (40)	
	Not acceptable	9 (22.5)	23 (57.5)	

Structural detail scores did not show a statistically significant difference between the groups, with most samples in both stained (90%) and unstained (75%) groups categorized under Score 3 (*p* = 0.070) (Table 2).

Acceptance levels showed that a higher proportion of stained samples were categorized as slightly acceptable (60%), whereas a larger proportion of unstained samples were rated as not acceptable (57.5%). This difference was statistically significant (*p* = 0.003) (Table 2).

### 3.3. Tooth Sections Under Compound Microscope

**Figure 4:**
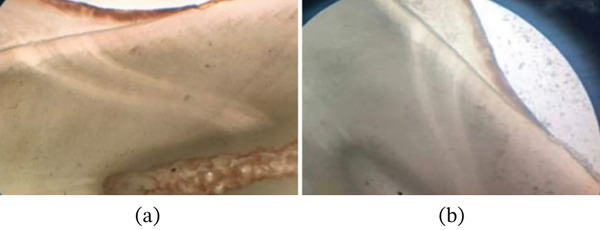
(a) Photomicrograph of *Terminalia chebula*‐stained tooth specimen under compound microscope. (b) Photomicrograph unstained tooth specimen under compound microscope.

Under compound microscopy, stained tooth sections (Figure 4a) demonstrated higher contrast, with 32 samples (80%) showing Grade 1 contrast compared with 19 samples (47.5%) in the unstained group (Figure 4b). This difference was statistically significant (*p* = 0.010) (Table 3).

**Table 3:** Comparison of contrast, structural details, and acceptance levels in tooth sections under compound microscope.

Parameter	Category	Stained *n* (%)	Unstained *n* (%)	*p* value
Contrast	Grade 1	32 (80)	19 (47.5)	0.01
	Grade 2	7 (17.5)	18 (45)	
	Grade 3	1 (2.5)	3 (7.5)	
Structural details	Score 1	19 (47.5)	8 (20)	0.018
	Score 2	19 (47.5)	25 (62.5)	
	Score 3	2 (5)	7 (17.5)	
Acceptance level	Highly acceptable	18 (45)	10 (25)	< 0.001
	Moderately acceptable	15 (37.5)	17 (42.5)	
	Slightly acceptable	6 (15)	10 (25)	
	Not acceptable	1 (2.5)	3 (7.5)	

Structural detail scores showed that 19 samples (47.5%) in the stained group demonstrated Score 1 compared with eight samples (20%) in the unstained group. The difference was statistically significant (*p* = 0.018) (Table 3).

Acceptance levels were higher in the stained group, with 18 samples (45%) rated as highly acceptable compared with 10 samples (25%) in the unstained group. This difference was statistically highly significant (*p* < 0.001) (Table 3).

### 3.4. Bone Sections Under Compound Microscope

**Figure 5:**
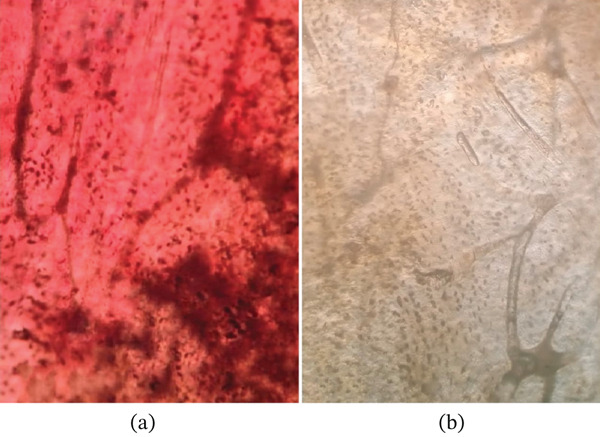
(a) Photomicrograph of *Terminalia chebula*‐stained bone specimen under compound microscope. (b) Photomicrograph unstained bone specimen under compound microscope.

Unstained bone sections (Figure 5b) demonstrated a higher proportion of Grade 1 (high‐contrast) samples compared with stained sections (Figure 5a) under compound microscopy (*p* < 0.001) (Table 4).

**Table 4:** Comparison of contrast, structural details, and acceptance levels in bone sections under compound microscope.

Parameter	Category	Stained *n* (%)	Unstained *n* (%)	*p* value
Contrast	Grade 1	4 (10)	11 (27.5)	0.01
	Grade 2	19 (47.5)	29 (72.5)	
	Grade 3	17 (42.5)	0 (0)	
Structural details	Score 1	6 (15)	8 (20)	0.376
	Score 2	27 (67.5)	29 (72.5)	
	Score 3	7 (17.5)	3 (7.5)	
Acceptance level	Highly acceptable	2 (5)	3 (7.5)	< 0.001
	Moderately acceptable	19 (47.5)	34 (85)	
	Slightly acceptable	15 (37.5)	3 (7.5)	
	Not acceptable	4 (10)	0 (0)	

Structural detail scores did not show a statistically significant difference between stained and unstained groups (**p = 0.376**) (Table 4).

Acceptance levels revealed that the majority of unstained samples were moderately acceptable (85%), whereas stained samples showed a wider distribution across categories. This difference was statistically significant (*p* = 0.001) (Table 4).

## 4. Discussion

Histological staining is fundamental in biomedical research and diagnostics, as it enhances visualization and interpretation of tissue microarchitecture [8]. Unlike decalcified sections, ground sections preserve mineral architecture but lack inherent contrast, necessitating the use of adjunctive staining methods.

The present study demonstrated that *T. chebula* significantly improved contrast and observer acceptance, particularly in tooth ground sections, highlighting its potential as a natural adjunct in hard tissue histology.

### 4.1. Effect on Contrast

The improved contrast observed in stained sections can be explained by the high tannin and polyphenolic content of *T. chebula*, which exhibits a strong affinity for proteinaceous components such as collagen in dentin and cementum. Polyphenols are known to bind to proteins through hydrogen bonding and precipitation reactions, thereby enhancing light absorption and optical differentiation [9].

Similar contrast enhancement has been reported with other plant‐based stains such as *Curcuma longa* and *Zingiber officinale*, which act through natural chromophores [10, 11]. However, in the present study, the effect was more pronounced in dental tissues, indicating a relatively higher affinity of the stain for enamel–dentin interfaces.

A key observation in this study was the superior staining performance in tooth sections compared with bone, along with a paradoxical finding under compound microscopy where unstained bone sections demonstrated relatively higher high‐contrast scores. This counter‐intuitive finding can be explained by structural and compositional differences between the tissues.

This can be attributed to structural and compositional differences between the tissues. Dentin contains an organized tubular architecture with a collagen‐rich matrix that facilitates dye binding and enhances contrast. In contrast, bone exhibits a more heterogeneous and variably mineralized structure, which may limit uniform stain penetration [12].

Additionally, bone ground sections inherently display natural contrast due to Haversian systems and lamellar organization. Under higher magnification, this intrinsic contrast may be sufficient, and additional staining may lead to over‐saturation or masking of fine details, thereby reducing perceived contrast. Similar inconsistencies in staining mineralized tissues using natural dyes have been reported previously [11].

Section thickness (~50 *μ*m) may have further influenced stain diffusion, particularly in bone, where denser areas can resist penetration. Earlier work by Brewer and Shellhamer [2] has emphasized that staining efficacy in ground sections is highly dependent on section thickness and permeability.

### 4.2. Effect on Structural Details

The ability to visualize fine structural details is essential for accurate histopathological assessment. Stained sections in this study, particularly tooth samples, revealed clearer demarcation of enamel, dentin, cementum, and incremental lines. This is in line with findings by Lakshmi et al. and Vinkhe et al. [5, 6], who demonstrated that plant‐derived stains can provide comparable or superior detail to routine dyes like hematoxylin and eosin. The improved visualization of cementum annulations and dentinal tubules may have potential relevance for applications such as age estimation; however, no forensic validation was performed in the present study [13, 14].

However, the enhancement in bone sections was less marked, which may be attributed to differences in mineral content and matrix composition between bone and dental tissues [12].. Similar limitations with plant‐based stains in bone have been reported previously [11]. Further optimization of staining protocols, such as increasing stain concentration or exposure time, may yield better results for bone specimens [15].

### 4.3. Observer Acceptance and Educational Utility

Observer acceptance was significantly higher for stained tooth sections, highlighting the practical value of *T. chebula* in improving interpretability. Enhanced clarity and contrast facilitate easier identification of histological landmarks, which is particularly beneficial in educational settings.

Natural stains have been proposed as cost‐effective and safer alternatives in histopathology laboratories. Studies by Ramamoorthy et al. and Mathur et al. have emphasized that plant‐derived stains reduce reliance on toxic chemicals such as xylene and synthetic dyes [16–18]. This makes them especially suitable for teaching laboratories and resource‐limited settings.

### 4.4. Green Chemistry and Safety Perspective

The use of *T. chebula* aligns with green chemistry principles, offering a biodegradable and less toxic alternative to conventional synthetic stains. Traditional histological chemicals, including certain dyes and clearing agents, have been associated with toxic and potentially carcinogenic effects, as well as environmental concerns [18]. Thus, the adoption of plant‐based stains may contribute to safer laboratory practices.

### 4.5. Medicinal Properties

*T. chebula* is well documented for its antioxidant, antimicrobial, and anti‐inflammatory properties [15, 19, 20]. However, their direct role in histological staining and tissue preservation was not evaluated in the present study.

### 4.6. Strength of the Study

A major strength of this study is the paired within‐specimen design, where each sample was evaluated before and after staining. This approach minimizes intersample variability related to differences in age, mineral composition, and anatomical variation, thereby increasing the reliability of the comparative findings.

### 4.7. Comparative Perspective

Other natural stains, such as those obtained from *Hibiscus rosa-sinensis*, beetroot, and *Bixa orellana*, have been explored for tissue staining with varying degrees of success. [10, 11, 18] However, direct comparison across studies remains challenging due to significant variability in staining concentration, duration of application, tissue type, and evaluation criteria. For instance, studies using *C. longa* and *Z. officinale* have reported moderate staining efficacy under differing methodological conditions, highlighting the lack of standardized protocols. [10, 11] In this context, the results of the present study position *T. chebula* as a promising alternative, particularly for dental tissues. Nevertheless, further controlled comparative studies are required to standardize staining parameters and establish its relative performance.

### 4.8. Limitations

The limitations of the study are listed as follows:

i. The present study did not assess the effect of staining duration on outcome. Previous reports indicate that both duration and concentration can significantly influence staining quality [11].
ii. The current protocol was not systemically optimized the concentration of *T. chebula* extract. Incremental adjustments could further enhance contrast and detail, particularly in bone sections. Standardization of stain concentration and pH may further improve reproducibility.
iii. The relatively lower enhancement in bone sections suggests a need for protocol refinement, possibly involving pretreatment modifications or enhanced stain penetration techniques [12].
iv. Variations in section thickness (~50 *μ*m), especially in bone specimens, may have influenced stain penetration and uniformity, thereby affecting staining outcomes.
v. The study did not include comparison with conventional stains for mineralized tissues, which limits direct evaluation of the relative efficacy of *T. chebula*.
vi. Interobserver agreement was not assessed, which may introduce subjective variability in grading despite blinded evaluation.

## 5. Conclusion and Future Directions

The findings of the present study suggest that *T. chebula* staining may enhance contrast and structural visualization in tooth ground sections, with high observer acceptance, indicating its potential applicability in teaching and research in dental histology. However, due to variable performance in bone sections and lack of diagnostic validation, its routine use in histopathology cannot currently be recommended. Compared with conventional stains, it may offer advantages such as cost‐effectiveness and reduced toxicity, although standardization remains limited. Its inconsistent performance in bone sections highlights tissue‐specific limitations. Further studies with standardized methodologies, larger sample sizes, and broader validation are required before routine implementation can be considered.

## Funding

This study was supported by the Rajiv Gandhi University of Health Sciences (10.13039/501100004255, UG23DEN227).

## Conflicts of Interest

The authors declare no conflicts of interest.

## Data Availability

The data that support the findings of this study are available from the corresponding author upon reasonable request.

## References

[1] Tan N. , Thentamil A. , and Jacob M. , Hard Tissue Architecture of Tooth - No Strain With Stain, Journal of Academy of Dental Education. (2017) 3, no. 1, 5–9, 10.18311/jade/2017/16448.

[2] Brewer H. E. and Shellhamer R. H. , Stained Ground Sections of Teeth and Bone, Stain Technology. (1956) 31, no. 3, 111–114, 10.3109/10520295609113787. 13324550

[3] Yadav S. M. , Wakode R. , Kumar S. , and Jadhav A. , Ground Sections of Teeth: Histopathological Study Modality, International Journal of Research in Medical Sciences. (2019) 7, no. 4, 1384–1387, 10.18203/2320-6012.ijrms20191359.

[4] Rath R. and Raghunath V. , Peels as an Alternative to Ground Sections – An In Vitro Microscopic Study, Journal of Oral and Maxillofacial Pathology. (2021) 25, no. 1, 31–36, 10.4103/jomfp.JOMFP_99_20, 34349408. 34349408 PMC8272485

[5] Lakshmi T. A. , Sumitra V. , and Victor R. , Application of MV10B Stain for Paraffin Sections of Teeth, International Journal of Health Sciences and Research. (2013) 3, no. 8, 17–21.

[6] Vikhe D. M. , Thete S. G. , Mastud C. S. , Mantri T. , Mhaske P. N. , Mastud S. P. , and Madanshetty P. , Staining the Ground Section of the Tooth Using an Innovative Plant Stain Found in the Pravara Region, India, Journal of Contemporary Dental Practice. (2020) 21, no. 10, 1113–1116. 33686031

[7] American Dental Association , Handling Extracted Teeth, 2003, American Dental Association, Available from: https://www.cdc.gov/dental-infection-control/hcp/dental-ipc-faqs/extracted-teeth.html.

[8] Kiernan J. A. , Histological and Histochemical Methods: Theory and Practice, 2015, 5th edition, Scion Publishing.

[9] Bag A. , Bhattacharyya S. K. , and Chattopadhyay R. R. , The Development of *Terminalia chebula* Retz. (Combretaceae) in Clinical research, Asian Pacific Journal of Tropical Biomedicine. (2013) 3, no. 3, 244–252, 10.1016/S2221-1691(13)60059-3, 23620847. 23620847 PMC3631759

[10] Lavanya A. , Sowmya S. V. , Rao Roopa S. , Augustine Dominic , and Haragannavar Vanishri C. , Natural Stain (Kumkum) Formulated by the Extract of Curcuma Aromatica and Slaked Lime in Histostaining of Oral Tissues: An Observational Study, Journal of Oral and Maxillofacial Pathology. (2021) 25, no. 1, 88–96. 34349417 10.4103/jomfp.JOMFP_90_20 PMC8272479

[11] Prabhu S. , Acharya S. , Muddapur M. et al., Efficacy of Natural Dyes in Hard Tissue Histology, J Oral Maxillofac Pathol.(2015) 19, no. 2, 188–195.26604495

[12] Habelitz S. , Marshall S. J. , Marshall G. W. , and Balooch M. , Mechanical Properties of Human Dental Enamel on the Nanometre Scale, Archives of Oral Biology. (2002) 47, no. 2, 125–133, 10.1016/S0003-9969(00)00089-3. 11163325

[13] Charles D. K. , Condon K. , Cheverud J. M. , and Buikstra J. E. , Cementum Annulation and Age Determination in Homo Sapiens. I. Tooth Variability and Observer Error, American Journal of Physical Anthropology. (1986) 71, no. 3, 311–320, 10.1002/ajpa.1330710306, 3812652. 3812652

[14] Shukla D. , Vinuth D. P. , Sowmya S. V. , Jeevan M. B. , Kale A. D. , and Hallikerimath S. , Cementum Made More Visual, Journal of Forensic Odonto-stomatology. (2012) 30, no. 1, 29–36, 23000809. PMC5734845 23000809

[15] Saxena S. , Lakshminarayan N. , Gudli S. , and Kumar M. , Anti Bacterial Efficacy of *Terminalia chebula*, Terminalia Bellirica, Embilica Officinalis and Triphala on Salivary Streptococcus Mutans Count - A Linear Randomized Cross Over Trial, Journal of Clinical and Diagnostic Research. (2017) 11, no. 2, ZC47–ZC51, 10.7860/JCDR/2017/23558.9355, 28384980. PMC5376913 28384980

[16] Sarbeen J. I. and Jayaraj G. , Light Microscopic Study of Cementum Under Different Histological Stains, Journal of Pharmaceutical Sciences and Research. (2015) 7, no. 9, 720–723.

[17] Ramamoorthy A. , Ravi S. , Jeddy N. , Thangavelu R. , and Janardhanan S. , Natural Alternatives for Chemicals Used in Histopathology lab: A Literature Review, Journal of Clinical and Diagnostic Research. (2016) 10, no. 11, EE01–EE04, 10.7860/JCDR/2016/23420.8860, 28050388. 28050388 PMC5198341

[18] Mathur S. , Mathur T. , Srivastava R. et al., Natural dyes: An Alternative to Synthetic Dyes in Histopathology, Journal of Oral Maxillofacial Pathology. (2021) 25, no. 1, 11–17.

[19] Cheng H. Y. , Lin T. C. , Yu K. H. , Yang C. M. , and Lin C. C. , Antioxidant and Free Radical Scavenging Activities of *Terminalia chebula* , Biological and Pharmaceutical Bulletin. (2003) 26, no. 9, 1331–1335, 10.1248/bpb.26.1331. 12951481

[20] Saleem A. , Husheem M. , Härkönen P. , and Pihlaja K. , Inhibition of Cancer Cell Growth by Crude Extract and the Phenolics of *Terminalia Chebula* Retz. Fruit, Journal of Ethnopharmacology. (2002) 81, no. 3, 327–336, 10.1016/S0378-8741(02)00099-5, 12127233. 12127233

